# Moxibustion-Simulating Bipolar Radiofrequency Suppresses Weight Gain and Induces Adipose Tissue Browning via Activation of UCP1 and FGF21 in a Mouse Model of Diet-Induced Obesity

**DOI:** 10.1155/2018/4737515

**Published:** 2018-09-10

**Authors:** Young Jun Koh, Ju-Hee Lee, Sung Yun Park

**Affiliations:** College of Korean Medicine, Dongguk University, 32 Dongguk-ro, Ilsandong-gu, Goyang-si, Gyeonggi-do 10326, Republic of Korea

## Abstract

**Background:**

Obesity is a pathological condition associated with various diseases including diabetes, stroke, arthritis, infertility, and heart disease. Moxibustion is widely used to prevent and manage obesity in traditional Asian medicine. We tested our hypothesis that moxibustion-simulating bipolar radiofrequency (M-RF) can suppress total body and white adipose tissue (WAT) weight gain via induction of WAT browning in a mouse model of diet-induced obesity (DIO).

**Methods:**

We designed an M-RF device that could accurately adjust the depth and temperature at which heat stimulation was administered into the abdomen of DIO mice. High-fat-fed male C57BL/6 mice were treated with the M-RF device every two or three days for three weeks. We then harvested WAT and serum from the mice and measured total body and WAT weight, size of adipocytes, mitochondrial contents, features of the dead adipocyte environment, and levels of uncoupling protein 1 (UCP1) and fibroblast growth factor 21 (FGF21).

**Results:**

Heat stimulation by M-RF in DIO mice resulted in precise temperature adjustment in the mice abdomen, with variance less than 1°C. Additionally, M-RF stimulation inhibited body and WAT weight gain, resulting in increased formation of beige adipocytes, increased mitochondrial content, and decreased formation of dead adipocytes in WAT. Moreover, treatment of M-RF induced expression of UCP1 and FGF21 in serum and/or epididymal WATs in DIO mice.

**Conclusion:**

Heat stimulation by M-RF treatment induced upregulation of UCP1 and FGF21 expression in serum and/or WATs, which was correlated with reduced total body and WAT weight gain in DIO mice.

## 1. Introduction

Moxibustion based on natural materials such as herbs is widely used in traditional Asian medicine clinics to prevent and manage various diseases such as polycystic ovary syndrome, ulcerative colitis, heart disease, irritable bowel syndrome, diabetic limbs, and obesity [1–6]. Previous animal studies showed that preventive moxibustion can also be used to adjust the levels of fat accumulation, blood lipids, and female sex hormones in rats and mice [6, 7]. Some researcher has proved that moxibustion leads to significant decrease of 5-hydroxytryptamine and adrenocorticotrophic hormone which play a role in decreasing blood flow into fat tissue in mouse [8]. However, moxibustion is limited in that it directly stimulates the outer layer of the skin with relatively high temperatures that may be difficult to adjust, and it generates smoke during use [9–11]. Thus, development of an effective, easy-to-use, and safe moxibustion system is needed.

Application of radiofrequency (RF)-induced thermal effects based on electrical systems is a minimally invasive treatment method used in various medical fields. Radiofrequencies are commonly used to treat tumors in the liver, lung, pancreas, and kidney, as well as to induce fat reduction and cellulite improvement [12–16]. Because RF is used to increase the temperature of a target point from 42°C to 46°C for hyperthermia therapy [17, 18], or for ablation therapy (in which temperature changes ranging from 50°C to 102°C are applied), skin can be simulated using RF devices to increase the temperature [10, 19].

Obesity accompanied by WAT growth poses serious concerns because it is closely related to the onset of several metabolic diseases, including type 2 diabetes, fatty liver disease, and cardiovascular diseases [20–22]. Recent studies have spurred interest in the antiobesity effects of WAT browning, which induces the characteristics of brown adipose tissue (BAT) in WATs outside of typical BAT locations [23]. Browning of adipocytes in WAT, which has genetic differences because of its distinct developmental origin from BAT [24], can be induced by various stimulations such as small molecules and environmental cues [25]. WAT browning occurs via several transcriptional factors, coregulators, lipid droplet-associated proteins, microRNAs, and growth factors, as well as mitochondrial uncoupling proteins [25–27].

Obesity is associated with chronic inflammation, which leads to various diseases such as diabetes, hypertension, and cardiovascular diseases [28]. Chronic inflammation in obesity is mainly caused by recruitment of inflammatory macrophages into the WATs, as well as by conversion of resident macrophages in the WATs [29, 30]. In the adipose tissue of obese individuals, macrophages with phagocytic activities surround and remove dead adipocytes in the WATs [29]. Therefore, it is important to characterize the adipose tissue in terms of macrophage infiltration and conversion status.

Obese patients who try losing weight via lifestyle interventions such as adjusting food intake and increasing the amount of physical exercise often fail to see significant improvements in their condition; moreover, pharmacotherapeutics aimed at managing obesity are often accompanied by side effects such as metabolic and psychologic diseases [31]. Thus, a novel mode of intervention that safely manages obesity and its related conditions is needed. In light of this unmet need, several trials have used not only medications, but also noninvasive medical devices to treat obesity [32]. Here, we describe the effects of moxibustion-simulating bipolar radiofrequency (M-RF) treatment on body weight reduction and adipocyte browning induction in a mouse model of diet-induced obesity (DIO).

## 2. Materials and Methods

### 2.1. Equipment

**Figure 1:**
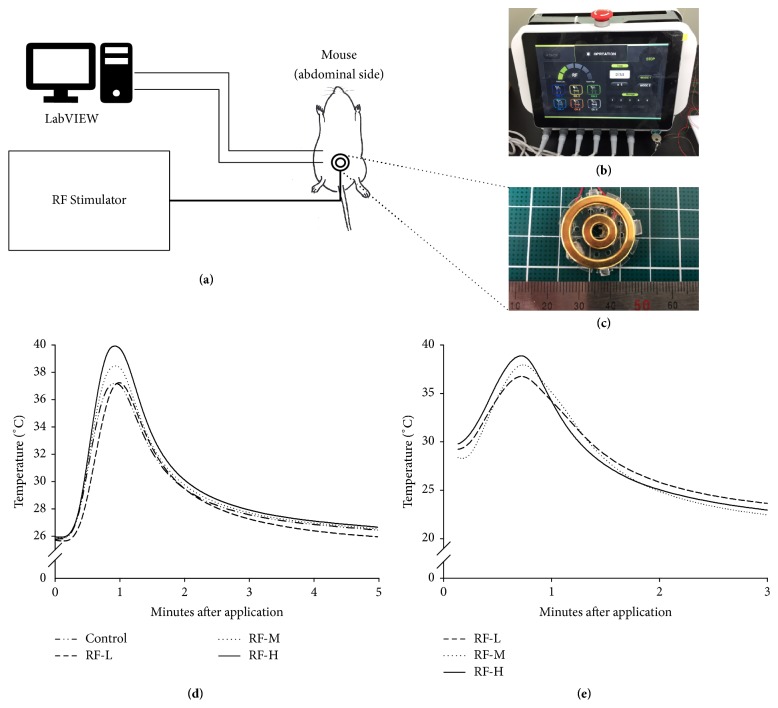
Development of the M-RF Stimulator. (a) Block diagram of the M-RF Stimulator and temperature measurement system. (b) Photo for front side of the M-RF Stimulator. (c) Bipolar electrode plate (5 mm, 1.8 mm, and 3 mm for diameters of the inner circle, outer circle, and width, respectively) in the M-RF Stimulator for use in mice. (d) Temperature change (maximum temperatures of 39.9°C, 38.4°C, 37.2°C, and 37.2°C for RF-H, RF-M, RF-L, and moxibustion (Moxibustion), respectively) in Yucatan pigs. (e) Temperature change (maximum temperatures of 38.8°C, 37.9°C, and 36.7°C for moxibustion, RF-H, RF-M, and RF-L, respectively) in mice.

To improve upon the method of classical moxibustion, we designed a bipolar RF device that is able to precisely control the depth and temperature at which stimulation is given, thereby allowing quantitative intervention. The main equipment consisted of two primary devices, an energy generator and a temperature measuring device (Figures 1(a) and 1(b)). A bipolar RF device mainly consisted of a power control board, display panel, and six bipolar probes. The power control board could generate a maximum power of 44.6 W with 50 Ω, 67.6 W with 200 Ω, and 50.8 W with 500 Ω, which was delivered to the subject through bipolar probes. The bipolar probes were circular (diameters of 5 mm, 1.8 mm, and 3 mm for the inner circle, outer circle, and width, respectively) and coated with Ag as a skin protectant (Figure 1(c)). The process was monitored and controlled by a display panel consisting of a 10.1′′ thin film transistor-liquid crystal display that had high resolution (1280*∗*800), which is standard for 4-wire touch screens. K-type temperature detecting needles were used to measure the temperatures at the surface of the abdomen and approximately 1 mm below the surface. The temperature data were sampled at 20 Hz and transmitted to the LabVIEW system (National Instruments, Austin, TX USA). The heating performance of the M-RF Stimulator was measured with skin samples of Yucatan pig and mice as shown in Figures 1(d) and 1(e). Each experimental procedure was conducted over a course of three minutes. The subjects were contained in plastic cube boxes (350 mm × 450 mm × 350 mm) maintained at 23°C ambient temperature and 50–60% humidity.

### 2.2. Mouse and Treatment

All animal experiments were approved by the Institutional Animal Care and Use Committee of Dongguk University (approval No. IACUC-2017-017-1). Eight-week-old male C57BL/6 mice were purchased from Central Lab Animal Inc. (Seoul, Korea). The mice were provided with* ad libitum* access to water and high-fat diet (HFD). The HFD (60% Kcal fat; Research Diets, New Brunswick, NJ) was provided for four weeks to induce diet-induced obesity (DIO). Afterwards, the mice were randomly distributed into the following four treatment groups: (1) no heat application (control,* n* = 10); (2) low-temperature stimulation (RF-L, 36.7°C,* n* = 10); (3) middle-temperature stimulation (RF-M, 37.9°C,* n* = 10); and (4) high-temperature stimulation (RF-H, 38.8°C,* n* = 10). We applied M-RF onto the abdominal skin of DIO mice at the three different temperatures for one minute per day every two days over three weeks while maintaining the HFD. At the end of the three-week application of M-RF, mice were anesthetized by intramuscular injection of a combination of anesthetics (mixture of tiletamine, zolazepam, and xylazine, each 10 mg/kg), weighed, and sacrificed. Blood was collected by cardiac puncture and centrifuged at 848 g for 15 minutes at 4°C, after which the resulting serum was harvested. The WAT specimens were immediately weighed and fixed in neutral buffered 4% formaldehyde for histochemical studies. The serum and WAT were stored at -80°C until further use.

### 2.3. Anesthesia and Euthanasia

Mice were anesthetized and euthanized by intramuscular injection of the combination of anesthetics (tiletamine-zolazepam 50mg/kg and xylazine 12 mg/kg) before RF treatment and histologic analysis.

### 2.4. Histologic and Morphometric Analysis

Mice were anesthetized by intramuscular injection of anesthetics, and WATs were fixed by cardiac perfusion of 1% paraformaldehyde in PBS and whole-mounted. To visualize the adipocytes and mitochondrial content in WATs, the tissues were incubated for four hours at room temperature with the following chemicals: 4,4-difluoro-4-bora-3a,4a-diaza-s-indacene (BODIPY) for adipocytes (1 *μ*g/ml in PBS; Invitrogen, Carlsbad, CA, USA) and MitoTracker Red CMXRos (MitoTracker) for mitochondrial contents (100 nM in PBS, Invitrogen). To verify the dead adipocytes, immunohistochemistry was performed as previously described [29, 33]. Briefly, the WATs were incubated for an hour at room temperature with blocking solution containing 5% whole donkey serum (Jackson ImmunoResearch Laboratories Inc., West Grove, PA, USA) in PBS-T (0.3% Triton X-100 in PBS). After blocking, the tissues were incubated overnight at room temperature while shaking with rat anti-mouse F4/80 antibody (clone Cl:A3-1, diluted 1:1,000; Serotec) in blocking solution to visualize infiltration of phagocytic macrophages in WATs, which is a characteristic of apoptotic adipocytes [29]. After five washes in PBS-T, whole-mounted tissues were incubated for four hours at room temperature with Cy3-conjugated anti-rat antibody (diluted 1:500; Jackson ImmunoResearch Laboratories Inc.) in blocking solution. For negative control experiments, primary or secondary antibodies were omitted during the immunohistochemistry process. All images were captured using a Nikon Eclipse Ts2 inverted fluorescent microscope (Nikon, Japan) equipped with high-definition color camera (DS-Qi2, Nikon) and then analyzed with the NIS Elements Imaging Software (version 4.30; Nikon). To calculate the adipocyte size, mitochondrial content, and UCP1 expression, stained images for BODIPY, MitoTracker, and UCP1 were captured and measured. To determine the adipocyte size in WATs, the diameters of adipocytes were measured in five randomly selected regions (~100 adipocytes per each region) per WAT. The mitochondrial content and expression of UCP1 were measured by analyzing the densities of the MitoTracker- or UCP1-positive areas based on the pixels in the regions of interest. During analysis, only pixels with an intensity of more than 50 were chosen to exclude background fluorescence. The number of beige adipocytes in five randomly selected regions (~100 adipocytes per each region) per WAT was counted. Dead adipocytes were detected using double-stained color images for BODIPY and F4/80. We counted the number of BODIPY^−^/clustered macrophage^+^ clustered regions for dead adipocytes in 10 randomly selected regions (~100 adipocytes per each region) per WAT in a blind manner.

### 2.5. Western Blotting

Total protein from WATs was extracted using a tissue homogenizer and lysis buffer (50 mM Tris-Cl, 150 mM NaCl, 1% 4-nonylphenyl-polyethylene glycol, 5 mM ethylenediaminetetraacetic acid, 1 M* threo*-1,4-Dimercapto-2,3-butanediol) containing protease inhibitor cocktail (GenDEPOT, Barker, TX, USA). The homogenates were centrifuged for 15 min at 15,928 g and 4°C. The supernatants were collected and centrifuged again, and the second supernatants were used for Western blotting analysis. To detect mouse FGF21 protein, protein-loaded membranes were first reacted with anti-FGF21 antibody (diluted 1:1000, Abcam, Cambridge, MA, USA) and then incubated with HRP-conjugated secondary antibody (diluted 1:5000, Thermo Scientific, Rockford, IL, USA). Finally, the FGF21 protein was detected with HRP substrate (Amersham Bioscience, Buckinghamshire, UK) using a Fusion FX7 chemiluminescence imaging system (Vilber Lourmat, France).

### 2.6. ELISA for Serum FGF21

To quantify FGF21 in mouse serum, an enzyme-linked immunosorbent assay (ELISA) was performed using the mouse FGF21 ELISA kit (R&D Systems, Minneapolis, MN, USA) according to the manufacturer's instructions. Briefly, mice were anesthetized by intramuscular injection of a combination of anesthetics, and 1 ml of whole blood was collected via cardiac puncture with 28-gauge syringe. The blood samples were then centrifuged for 15 minutes at 848 g and 4°C, after which the resulting serum samples were stored at -80°C until analysis.

### 2.7. Statistics

Values are presented as the means ± standard deviation (SD). Statistical analyses consisted of one-way analysis of variance (ANOVA) followed by Tukey's multiple comparison or a Student's t-test. P values < 0.05 were considered statistically significant. All experiments were performed independently at least three times, and the data were analyzed using the Prism 5.0 software (GraphPad Software, Inc., San Diego, CA, USA).

## 3. Results

### 3.1. Thermal Stimulation Using an M-RF Stimulator

To evaluate the performance of the M-RF Stimulator, we measured the temperature of Yucatan pigs and mice. The temperature curves of the M-RF Stimulator (39.9 ± 0.12°C, 38.4 ± 0.12°C, and 37.2 ± 0.13°C (mean ± SD) for maximum temperatures of RF-H, RF-M, and RF-L, respectively) showed patterns similar to those of moxibustion (37.2 ± 0.21°C maximum temperature) at 2 mm from the skin of Yucatan pigs (Figure 1(d)) and at 1 mm from the skin of mice (38.8°C, 37.9°C, and 36.7°C for RF-H, RF-M, and RF-L, respectively) (Figure 1(e)). The average times required for the temperature to increase from approximately 30°C to the maximum temperature for each temperature curve in the Yucatan pig experiment were 17.4 sec, 16.8 sec, 18.9 sec, and 13.3 sec for RF-H, RF-M, RF-L, and moxibustion, respectively (Figure 1(d)).

### 3.2. Thermal Stimulation by M-RF Suppresses Body and WAT Weight Gain in DIO Mice

**Figure 2:**
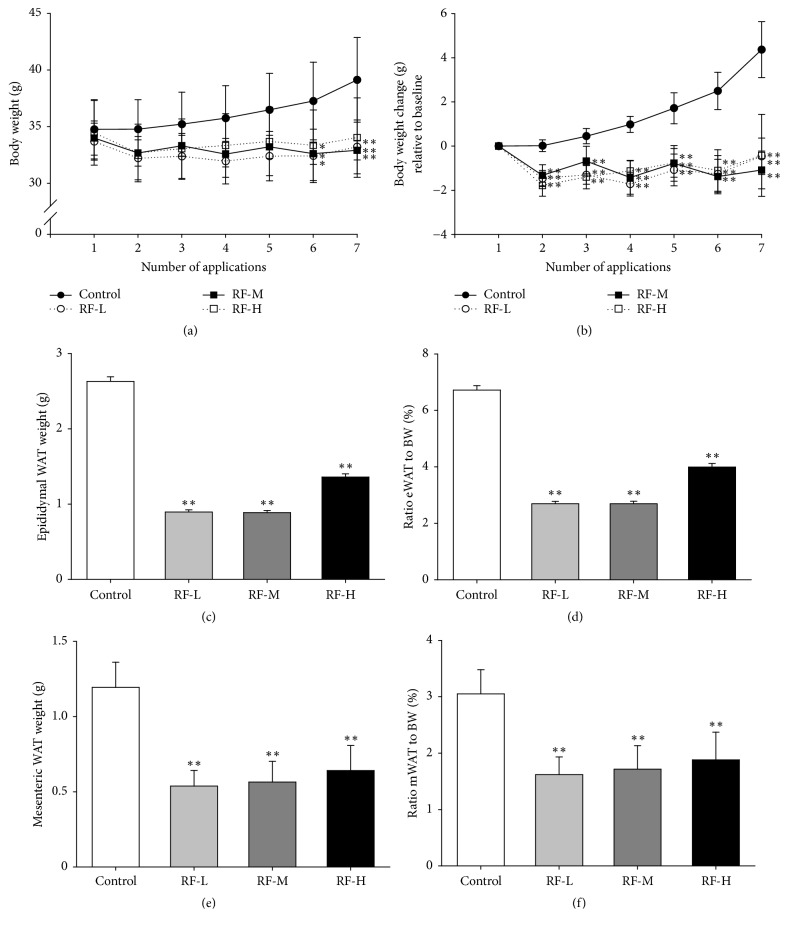
M-RF stimulation suppresses total body and WAT weight gain in DIO mice. (a) Body weights (g) of control (*n *= 10), RF-L (*n *= 10), RF-M (*n *= 10), or RF-H (*n *= 10) groups measured before M-RF stimulation. (b) Body weight changes (g) relative to the base level (the day of first application) in each group (*n *= 10). (c, e) Weights (g) of WATs in each group (*n *= 10). (d, f) Ratio of eWAT (epididymal WAT) weight (d) and mWAT (mesenteric WAT) (f) to body weight at the end-points of the experiment in each group (*n *= 10). Results are presented as the means ± SD. *∗*, P < 0.05 versus control. *∗∗*, P < 0.01 versus control.

To determine the effects of M-RF-induced heat stimulation on body weight gain in DIO mice (control), we applied M-RF on DIO mice at three levels of temperature, low (36.7°C; F-L), middle (37.9°C; RF-M), and high (38.8°C; RF-H), for three weeks (three min per day every two days). At three weeks after the start of M-RF treatment, total body weight measurements of the control group that received no heat stimulation had significantly increased (39.13 ± 3.736 g,* n* = 10), whereas those of RF-L (33.22 ± 2.371 g,* n* = 10, P < 0.01 versus control), RF-M ( 32.92 ± 0.8585 g,* n* = 10, P < 0.01 versus control), and RF-H (34.04 ± 3.505 g,* n* = 10, P < 0.01 versus control) had not increased significantly (Figure 2(a)). Moreover, in contrast with the control (at end of the experiment; 4.367 ± 1.268 g,* n* = 10), all three levels of M-RF stimulation groups showed changes in body weight relative to the day of first application (RF-L, -0.46 ± 0.826 g,* n* = 10, P < 0.01 versus control; RF-M, -1.08 ± 0.858 g,* n* = 10, P < 0.01 versus control; RF-H, -0.42 ± 1.85 g,* n* = 10, P < 0.01 versus control) (Figure 2(b)). Consistently, weights of epididymal and mesenteric WATs in M-RF-treated DIO mice did not significantly increase in all three levels (RF-L, 0.895 ± 0.0284 g,* n* = 10, P < 0.01 eWAT versus control and 0.538 ± 0.1039 g,* n* = 10, P < 0.01 mWAT versus control; RF-M, 0.8866 ± 0.0286 g,* n* =10, P < 0.01 eWAT versus control and 0.565 ± 0.1373 g,* n* = 10, P < 0.01 mWAT versus control; RF-H, 1.359 ± 0.0427 g,* n* = 10, P < 0.01 eWAT versus control and 0.641 ± 0.1664 g,* n* = 10, P < 0.01 mWAT versus control) compared to the control (2.630 ± 0.0610 gram,* n* = 10 for eWAT and 1.194 ± 0.1673 g,* n* = 10 for mWAT) (Figures 2(c) and 2(e)). Moreover, ratios of WAT-to-body weight were lower in RF-L (2.69 ± 0.085%,* n* = 10, P < 0.01 eWAT versus control and 1.62 ± 0.313%,* n* = 10, P < 0.01 mWAT versus control), RF-M (2.69 ± 0.087%,* n* = 10, P < 0.01 eWAT versus control and 1.72 ± 0.417%,* n* = 10, P < 0.01 mWAT versus control), and RF-H groups (3.99 ± 0.125%,* n* = 10, P < 0.01 eWAT versus control and 1.88 ± 0.489%,* n* = 10, P < 0.0101 mWAT versus control) than in the control (6.72 ± 0.156%,* n* = 10 for eWAT and 3.05 ± 0.428%,* n* = 10 for mWAT) (Figures 2(d) and 2(f)).

### 3.3. Thermal Stimulation by M-RF Decreases the Size of Adipocytes and Increases Mitochondrial Contents in the WATs of DIO Mice

**Figure 3:**
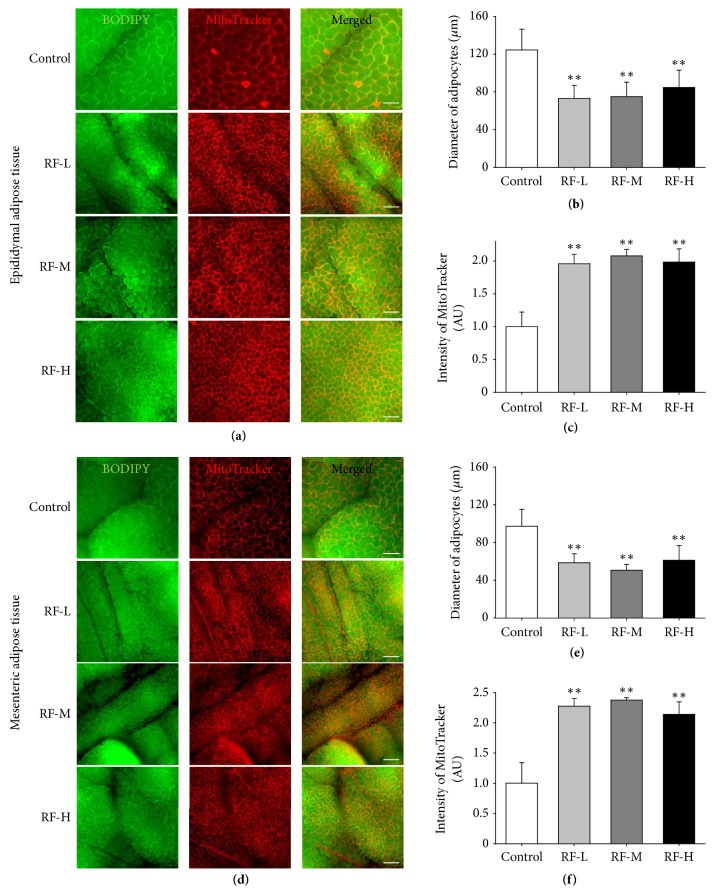
M-RF stimulation reduces the size of adipocytes and increases mitochondrial contents in the WATs of DIO mice. (a, d) Whole-mounted WATs stained with BODIPY (green) and MitoTracker (red). Note that all three levels of M-RF stimulation resulted in decreases in the size of adipocytes and increases in the mitochondrial contents compared to the control. Scale bar, 200 *μ*m. (b, c, e, and f) Diameter of adipocytes and density of MitoTracker-positive area were measured in five randomly selected regions (~100 adipocytes per region) per WAT in control (*n *= 10), RF-L (*n *= 10), RF-M (*n *= 10), and RF-H (*n *= 10) and presented as micrometers for diameter of adipocytes and as an arbitrary unit (AU) for the ratio of the pixel densities compared to the control for intensity of MitoTracker. Results are presented as the means ± SD. *∗∗*, P < 0.01 versus control.

To describe the cellular changes in WATs induced by M-RF stimulation, we harvested the epididymal and mesenteric WATs from the M-RF-treated DIO mice and observed the size and mitochondrial contents of adipocytes in both WATs using whole-mounted immunostaining (Figures 3(a) and 3(d)). In the control group, the average diameter of the adipocytes was 124.3 ± 22.14 *μ*m in epididymal (*n* = 5,000 cells) and 97.21 ± 17.89 *μ*m in mesenteric WATs (*n* = 5,000 cells) (Figures 3(b) and 3(e)). In M-RF-treated groups, the sizes of adipocytes in both epididymal and mesenteric WATs were significantly smaller (73.19 ± 13.56 *μ*m,* n* = 5,000 cells, P < 0.01 eWAT versus control and 58.60 ± 9.446 *μ*m,* n* = 5,000 cells, P < 0.01 mWAT versus control), RF-M (75.03 ± 15.25 *μ*m,* n* = 5,000 cells, P < 0.01 eWAT versus control and 50.57 ± 6.22 *μ*m,* n* = 5,000 cells, P < 0.01 mWAT versus control), and RF-H (84.61 ± 18.48 *μ*m,* n* = 5,000 cells, P < 0.01 eWAT versus control and 61.08 ± 15.78 *μ*m,* n* = 5,000 cells, P < 0.01 mWAT versus control) (Figures 3(b) and 3(e)). Furthermore, contents of mitochondria in the adipocytes of epididymal and mesenteric WATs were increased by all three levels of M-RF stimulation (RF-L, 1.959 ± 0.1447 AU,* n* = 5,000 cells, P < 0.01 eWAT versus control and 2.275 ± 0.1275 AU,* n* = 5,000 cells, P < 0.01 mWAT versus control; RF-M, 2.077 ± 0.0999 AU,* n* = 5,000 cells, P < 0.01 eWAT versus control and 1.983 ± 0.2004 AU,* n* = 5,000 cells, P < 0.01 mWAT versus control; RF-H, 1.983 ± 0.2004 AU,* n* = 5,000 cells, P < 0.01 eWAT versus control and 2.141 ± 0.2071 AU,* n* = 5,000 cells, P < 0.01 mWAT versus control) (Figures 3(c) and 3(f)).

### 3.4. Thermal Stimulation by M-RF Induces Expression of UCP1 and Formation of Beige Adipocytes in the WATs of DIO Mice

**Figure 4:**
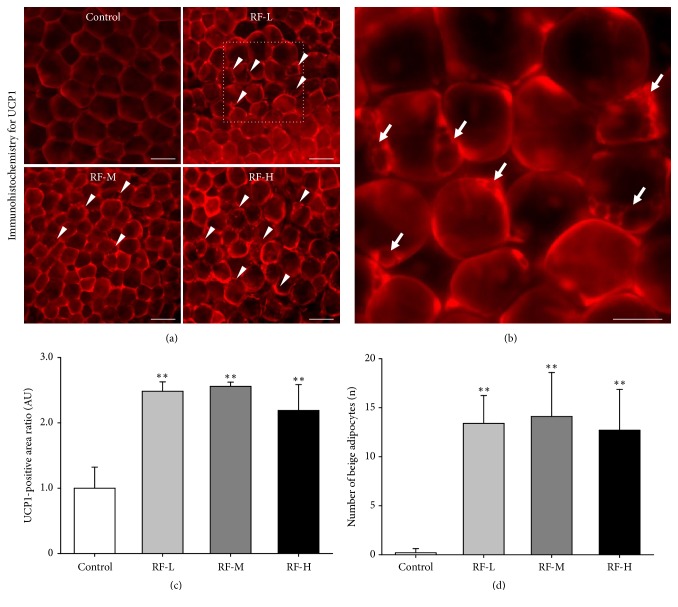
M-RF stimulation increases UCP1 expression and formation of beige adipocytes in the WATs of DIO mice. (a) Whole-mounted WATs immunostained for UCP1 (red). Scale bar, 100 *μ*m. (b) Higher magnification image of RF-L in (a) showing characteristic beige adipocytes. Scale bar, 50 *μ*m. Note that all three levels of M-RF stimulation increased UCP1 expression and formation of beige adipocytes compared to control. Scale bar, 50 *μ*m. (c, d) Ratio of UCP1-positive areas compared to control (c) and numbers of beige adipocytes (d) calculated in five randomly selected regions (~100 adipocytes per each region) per WAT in control (*n *= 10), RF-L (*n *= 10), RF-M (*n *= 10), and RF-H (*n *= 10). Results are presented as means ± SD. *∗∗*, P < 0.01 versus control.

To identify the underlying mechanism of increased mitochondrial contents induced by M-RF treatment in DIO mice, we analyzed UCP-1 expression in epididymal WATs because activation of UCP-1 is a component of mitochondrial activation [34] that is responsible for browning of WATs [25]. Similar to the changes in mitochondrial contents, UCP-1 was highly expressed in the epididymal WATs following RF-L (2.438 ± 0.1440 AU,* n* = 5,000 cells, P < 0.01 versus control), RF-M (2.558 ± 0.0646 AU,* n* = 5,000 cells, P < 0.01 versus control), and RF-H (2.189 ± 0.3965 AU,* n* = 5,000 cells, P < 0.01 versus control) (Figures 4(a) and 4(c)). Consistent with previous reports [33], while beige adipocytes were rarely observed in the WATs of control DIO mice (0.200 ± 0.421,* n* = 5,000 cells), large numbers of beige adipocytes with small droplets were observed in the eWATs of DIO mice that received RF-L (13.4 ± 2.84,* n* = 5,000 cells, P < 0.01 versus control), RF-M (14.1 ± 4.48,* n* = 5,000 cells, P < 0.01 versus control), and RF-H (12.7 ± 4.16,* n* = 5,000 cells, P < 0.01 versus control) stimulation (Figures 4(b) and 4(d)).

### 3.5. Thermal Stimulation by M-RF Inhibits Infiltration of Macrophages into Dead Adipocytes in the WATs of DIO Mice

**Figure 5:**
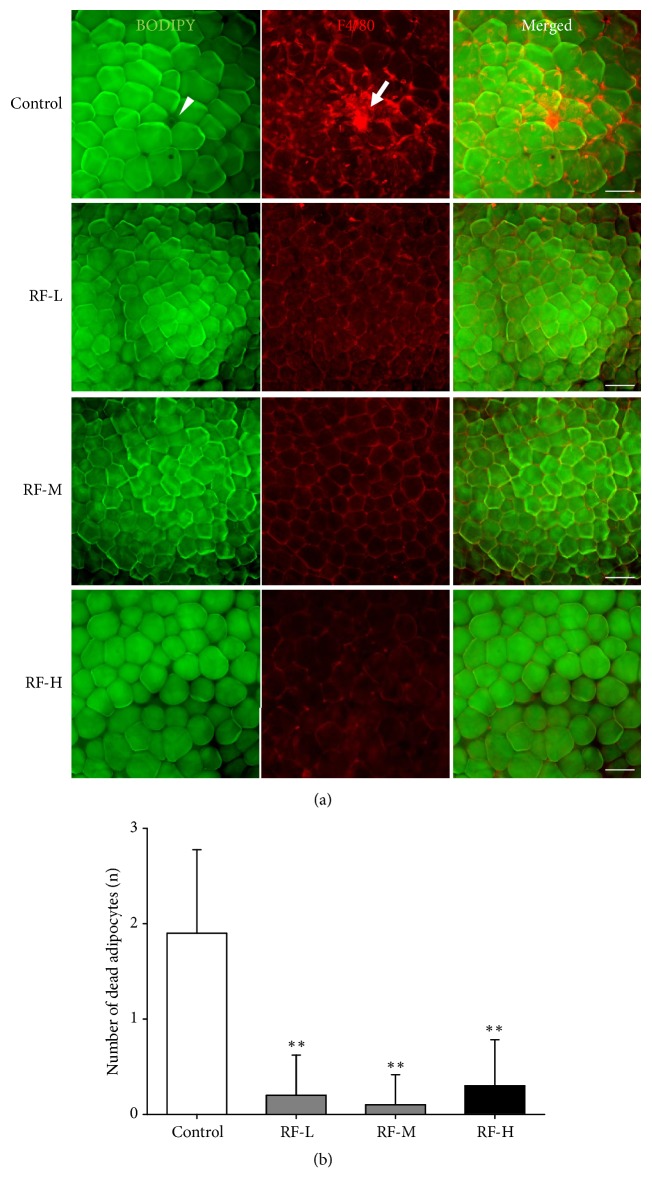
M-RF stimulation suppresses dead adipocyte formation in the WATs of DIO mice. (a) Whole-mounted WATs double-stained with BODIPY (green) and F4/80 (red). Note that all three levels of M-RF stimulation resulted in decreased dead adipocyte formation compared to control. Scale bar, 100 *μ*m. (b) Number of BODIPY^−^/clustered F4/80^+^ dead adipocytes counted in 10 randomly selected regions (~100 adipocytes per region) per WAT in control (*n *= 10), RF-L (*n *= 10), RF-M (*n *= 10), and RF-H (*n *= 10). Results are presented as the means ± SD. *∗∗*, P < 0.01 versus control.

To investigate the presence of dead adipocytes and macrophage infiltration into WATs, whole-mounted WATs were costained with BODIPY (neutral lipid binding chemical) and F4/80 (phagocytic macrophage marker). In control mice, we observed dead adipocytes with macrophage clumps (BODIPY^−^/F4/80^+^ cells) in their epididymal WATs (1.9 ± 0.88,* n* = 5,000 cells, P < 0.01), but these were not found in their mesenteric WATs (data not shown) (Figure 5(a)). In contrast, we rarely detected BODIPY^−^/F4/80^+^ dead adipocytes in the epididymal WATs of RF-L (0.20 ± 0.42,* n* = 5,000 cells, P < 0.01 versus control), RF-M (0.10 ± 0.32,* n* = 5,000 cells, P < 0.01 versus control), and RF-H (0.30 ± 0.48,* n* = 5,000 cells, P < 0.01 versus control) groups (Figure 5(b)).

### 3.6. Thermal Stimulation by M-RF Increases the Expression of FGF21 in Both Serum and WATs of DIO Mice

**Figure 6:**
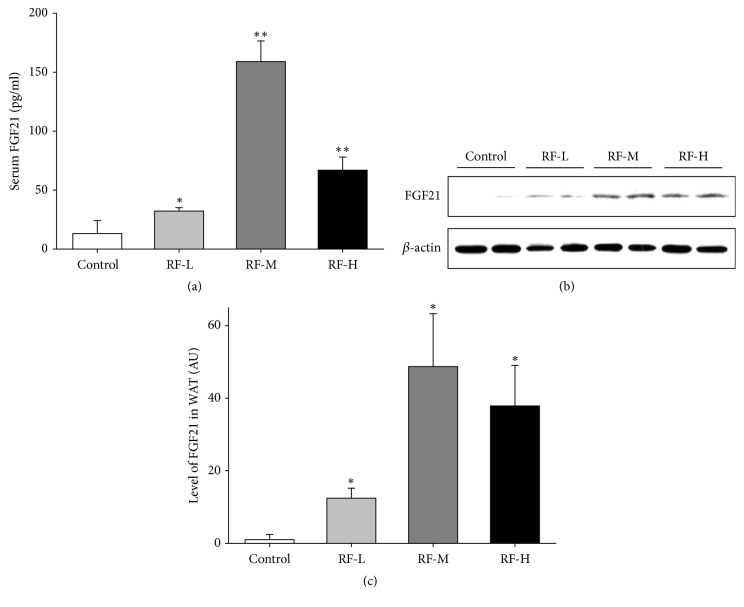
M-RF stimulation increases the level of FGF21 in serum and WAT of DIO mice. (a) ELISA for serum FGF21 in control (*n *= 5), RF-L (*n *= 5), RF-M (*n *= 5), and RF-H (*n *= 5). (b) Representative Western blotting for FGF21 in WATs of DIO mice in control (*n *= 5), RF-L (*n *= 5), RF-M (*n *= 5), and RF-H (*n *= 5). (c) Quantification of (b) and calculated in terms of ratio relative to control. Note that all three levels of M-RF stimulation significantly increased the level of FGF21 in serum and WAT compared to the control. Results are presented as the mean ± SD. *∗*, P < 0.05 versus control. *∗∗*, P < 0.01 versus control.

FGF21 has favorable effects in several metabolic diseases including type 2 diabetes, dyslipidemia, and obesity. We analyzed the level of FGF21 from serum and epididymal WATs from DIO mice and found that M-RF stimulation increased serum FGF21 levels in RF-L (32.15 ± 2.839,* n* = 5, P < 0.05 versus control), RF-M (159.0 ± 17.43,* n* = 5, P < 0.01 versus control), and RF-H (66.81 ± 11.25,* n* = 5, P < 0.01 versus control) groups, whereas the control group showed a significantly lower amount of serum FGF21 (13.14 ± 10.92,* n* = 5) (Figure 6(a)). Similarly, FGF21 protein expression was increased in the epididymal WATs in response to all three levels of M-RF stimulation (RF-L, 12.45 ± 2.730 AU,* n* = 5, P < 0.05 versus control; RF-M, 48.78 ± 14.54 AU,* n* = 5, P < 0.05 versus control; RF-H, 37.94 ± 11.15 AU,* n* = 5, P < 0.05 versus control) (Figures 6(b) and 6(c)).

## 4. Discussion

We manufactured a moxibustion-simulating heat stimulation device using bipolar RF and showed that our bipolar RF was effective for precise adjustment of depth and temperature of thermal stimulation targeting abdominal WAT, which provides an advantage over monopolar RF devices. Our results demonstrated that thermal stimulation by M-RF treatment induced profound reductions in total body and WAT weight, which were accompanied by adipose tissue browning and elevation of UCP1 and FGF21 expression in DIO mice.

Tissue heating by RF stimulation occurs in response to the induction of ion currents into target tissue by applying an RF wave field between an electrode plate and a ground plate. While the two plates of monopolar RF are located on opposing sides, the two plates of bipolar RF are positioned closely on the same surface. Studies have shown that by changing the power of an RF generator makes it easy to precisely increase the temperature by depth in bipolar RF compared with monopolar RF [35, 36]. Because of these advantages, bipolar RF is used in various medical fields for tumor treatment and fat reduction [12, 13, 37].

Our findings show that heat stimulation by RF treatment leads to beige adipocyte induction in DIO mice, which is interesting because beige adipocyte induction in WATs is a key feature of cold exposure [25]. Beige adipocyte induction can be promoted by several external stimuli, including *β*_3_-adrenergic receptor agonist, A_2A_ agonist, thiazolidinedione, miRNA 155, and cold exposure [25, 38–40], which exert their effects by activating peroxisome proliferator-activated receptor gamma, peroxisome proliferator-activated receptor gamma coactivator-1 alpha (PGC-1*α*), *β*_3_-adrenergic receptor, and adenosine receptor in WAT and other tissues [25, 38–40]. Specifically, cold exposure is a strong inducer of beige adipocyte induction that functions by increasing mitochondrial biogenesis [25]; however, the detailed mechanism responsible for beige adipocyte induction by heat stimulation has yet to be clearly identified. A previous study indicated that heat stimulation by RF may result in fat reduction and cellulite improvement and that heat stimulation may also cause minor inflammation and promote collagen formation [15]. Another report suggested a unique effect of bipolar RF in enhancing fat metabolism, which may contribute to treating cellulite. Because bipolar RF can penetrate to depths of more than 3 mm in depth, heat stimulation by bipolar RF can alter the surrounding adipose tissue by inducing thermal damage [41]. Thus, it is possible that heat stimulation by the M-RF device that we designed is strong enough to cause browning of WAT and lead to reduced total body weight.

The present findings also show that treatment with RF inhibits the infiltration of macrophages into dead adipocytes in WATs, which is a characteristic inflammatory phenotype in obesity. Obesity can induce chronic inflammation, thereby leading to onset of various diseases such as diabetes, hypertension, and cardiovascular diseases [28]. In obese individuals, phagocytic macrophages can be recruited into WATs from the bone marrow, or resident macrophages in WATs may be converted into phagocytic macrophages [29, 30]. Such changes in the macrophage environment lead to production of inflammatory cytokines such as interleukin-1*β*, monocyte chemoattractant protein-1, and tumor necrosis factor-*α* [28]. Therefore, it is possible that the decrease in the number of dead adipocytes and inhibition of macrophage infiltration in WATs are responsible for the weight reduction caused by RF stimulation.

Our results showed that heat stimulation by RF treatment induces profound mitochondrial biogenesis and expression of UCP1 in WATs of DIO mice. Browning of WATs is accompanied by generation of mitochondria and expression of related genes in the WATs [42]. During browning through mitochondrial biogenesis in WATs, activation of PGC-1*α*, peroxisome proliferator-activated receptor gamma, PR domain containing 16, and other coactivators induce the expression of mitochondria-related genes including UCP1 [23]. UCP1 is mitochondrial carrier protein expressed brown adipocytes that are responsible for generating heat through proton transport via ATP synthesis [43, 44]. Particularly, UCP1 plays crucial roles in the development of BAT and the production of beige adipocytes from white adipocytes, even outside typical BAT locations [23]. Expression of adipocyte-specific UCP1 leads to prevention of obesity by modulating mitochondrial membrane potential [45]. Conversely, deletion of UCP1 induces obesity by abolishing diet-induced thermogenesis in mice kept at thermoneutral temperature, regardless of the type of diet (normal chow, high-fat) [46]. Although the mechanism underlying RF stimulation-induced UCP1 expression and adipocyte browning in WAT remains unclear, our results demonstrated that browning can occur in the WATs of DIO mice upon RF stimulation.

A clinical study of twelve healthy males using a temperature-controlled water bath showed that levels of serum FGF21 and free fatty acids increased after immersing their lower legs in hot water at 39°C, 42°C, or 43°C [47]. Moreover, heat treatment and activation of heat shock proteins have been shown to improve insulin sensitivity of skeletal muscle, as well as to reduce plasma triglyceride and free fatty acids of genetically obese mice in conjunction with a decrease in WAT mass [48]. Traditionally, only cold exposure was associated with formation of beige adipocytes [24, 25, 42]; however, previous reports have shown that heat stimulation can also induce beige adipocyte formation [15, 47], which led us to test the effects of RF heat stimulation in WAT browning. Consistent with the results of a previous report [47], our results demonstrated that heat stimulation by RF treatment can induce elevation of FGF21 in the serum and WATs. FGF21 has diverse metabolic effects that are beneficial for managing obesity, increasing fatty acid oxidation in the liver, and improving insulin sensitivity in obese individuals [49, 50]. Recent studies have shown that elevation of adipose-derived and serum FGF21 levels lead to upregulation of UCP1 transcription in BAT and WAT [51–53]. Moreover, several* in vitro* and* in vivo* studies demonstrated that high concentrations of serum FGF21 cause browning of WATs and are positively correlated with nonshivering thermogenesis. Furthermore, FGF21 increases the expression of cell death activator CIDE-A, carnitine palmitoyltransferase-1 beta, and UCP1 in both brown and white adipocytes in a PGC-1*α*-dependent manner [51]. FGF21 also plays central roles in activation of the sympathetic nerve system and increases in energy expenditure, which are correlated with weight loss [54]. Moreover, FGF21 has been found to increase CCL11 expression to recruit eosinophils into WAT, where CCL11 leads to infiltration of macrophages and generation of beige adipocytes from adipocyte precursors [55]. Thus, we assume that M-RF treatment in DIO mice induced the expression of FGF21 and UCP1, which in turn influenced adipocyte browning by activation of the sympathetic nerve and changes in the commitment of adipocyte precursors.

## 5. Conclusions

Building upon the idea of moxibustion, we manufactured an M-RF heat stimulation device and demonstrated its antiobesity effects in DIO mice. Heat stimulation using M-RF effectively suppressed body and WAT weight gain while increasing the formation of beige adipocytes, mitochondrial content, and higher expression of UCP1 and FGF21 in DIO mice.

## Data Availability

The data used to support the findings of this study are available from the corresponding author upon request.
